# The Impact of Telemedicine Visits on the Controlling High Blood Pressure Quality Measure During the COVID-19 Pandemic: Retrospective Cohort Study

**DOI:** 10.2196/32403

**Published:** 2022-03-23

**Authors:** Siqin Ye, D Edmund Anstey, Anne Grauer, Gil Metser, Nathalie Moise, Joseph Schwartz, Ian Kronish, Marwah Abdalla

**Affiliations:** 1 Department of Medicine Columbia University Irving Medical Center New York, NY United States; 2 Center for Behavioral Cardiovascular Health Columbia University Irving Medical Center New York, NY United States; 3 Department of Psychiatry and Behavioral Sciences Stony Brook University Stony Brook, NY United States

**Keywords:** telemedicine, hypertension, blood pressure, quality of care, impact, COVID-19, cohort, cardiology, telehealth, retrospective

## Abstract

**Background:**

Telemedicine visit use vastly expanded during the COVID-19 pandemic, and this has had an uncertain impact on cardiovascular care quality.

**Objective:**

We sought to examine the association between telemedicine visits and the failure to meet the *Controlling High Blood Pressure* (BP) quality measure from the Centers for Medicare & Medicaid Services.

**Methods:**

This was a retrospective cohort study of 32,727 adult patients with hypertension who were seen in primary care and cardiology clinics at an urban, academic medical center from February to December 2020. The primary outcome was a failure to meet the *Controlling High Blood Pressure* quality measure, which was defined as having no BP recorded or having a last recorded BP of ≥140/90 mm Hg (ie, poor BP control). Multivariable logistic regression was used to assess the association between telemedicine visit use during the study period (none, 1 telemedicine visit, or ≥2 telemedicine visits) and poor BP control; we adjusted for demographic and clinical characteristics.

**Results:**

During the study period, no BP was recorded for 2.3% (486/20,745) of patients with in-person visits only, 27.1% (1863/6878) of patients with 1 telemedicine visit, and 25% (1277/5104) of patients with ≥2 telemedicine visits. After adjustment, telemedicine use was associated with poor BP control (1 telemedicine visit: odds ratio [OR] 2.06, 95% CI 1.94-2.18; *P*<.001; ≥2 telemedicine visits: OR 2.49, 95% CI 2.31-2.68; *P*<.001; reference: in-person visits only). This effect disappeared when the analysis was restricted to patients with at least 1 recorded BP (1 telemedicine visit: OR 0.89, 95% CI 0.83-0.95; *P*=.001; ≥2 telemedicine visits: OR 0.91, 95% CI 0.83-0.99; *P*=.03).

**Conclusions:**

Increased telemedicine visit use is associated with poorer performance on the *Controlling High Blood Pressure* quality measure. However, telemedicine visit use may not negatively impact BP control when BP is recorded.

## Introduction

The COVID-19 pandemic resulted in the rapid expansion of telemedicine as an integral part of outpatient care [1-7]. A telemedicine visit is defined as using video and telephone technology to connect patients to their clinicians as a substitute for in-person office visits. Telemedicine visits have helped maintain the continuity of care and preserve patients’ access to care and are expected to remain a significant part of the health care delivery landscape even after the COVID-19 pandemic ends [8,9]. However, despite its increased utilization, concern has arisen that the content of care delivered via telemedicine visits may differ from that delivered during in-person visits. In particular, studies have shown that telemedicine visits are less likely to address standard components of care, such as blood pressure (BP) assessment, laboratory testing, and medication prescriptions and test orderings [10,11].

BP measurement is a fundamental component of hypertension management [12,13], and there has been increasing concern that the increased use of telemedicine visits in primary care and cardiology outpatient settings may impact the accurate assessment of BP and, in turn, how well BP is controlled for patients with hypertension [14]. To address this potentially unintended consequence of telemedicine expansion, we performed an electronic health record (EHR)–based retrospective analysis of a diverse population of patients with hypertension receiving care at a large, urban, academic medical center across an 11-month period during the COVID-19 pandemic. Using the specification of the *Controlling High Blood Pressure* quality measure from the Centers for Medicare & Medicaid Services (CMS) that defines poor BP control as having no recorded BP measurements or having a last recorded BP of ≥140/90 mm Hg [15,16], we sought to determine the association between telemedicine visit use and the failure to meet this widely used measure for benchmarking population-level quality of care, hypothesizing that the increased use of telemedicine visits can lead to poorer performance on this quality measure.

## Methods

### Ethics Approval

The study was approved by the Columbia University Irving Medical Center Institutional Review Board (AAAT0375), in accordance with the ethical standards of the responsible committee on human experimentation (institutional and national) and with the Helsinki Declaration of 1975, as revised in 2000. Informed consent was waived due to the study being of minimal risk.

### Study Design

After approval by our institutional review board, we queried our EHR (Epic EHR; Epic Systems) to identify all completed outpatient visits to primary care and cardiology clinics at Columbia University Irving Medical Center from February 1, 2020, to December 31, 2020. The Medical Center transitioned to Epic EHR on February 1, 2020, that is, 1 month prior to the start of the COVID-19 pandemic in New York City. For each visit, we extracted information, including patients’ date of birth, sex, race, and ethnicity; the primary payer; and the International Classification of Diseases, 10th Revision (ICD-10) codes associated with the visits. BP data associated with the visits were extracted from Epic flow sheets, and BP measurements were recorded in accordance with standard clinical protocols (ie, staff or clinicians recorded BP measurements in the office during in-person visits, and self-reported BP values that were measured at home during telemedicine visits at clinicians’ discretion).

For our analysis, we defined the study population by using specifications published by the CMS for the 2020 version of the quality measure *HTN-2: Controlling High Blood Pressure* [16]. Specifically, we included all patients aged between 18 and 85 years with a diagnosis of hypertension, which was defined as having the ICD-10 code “E10” associated with any visit during the study period. Patients with end-stage renal disease, those with a history of kidney transplant, or those who were pregnant during the study period were excluded based on the ICD-10 codes for these conditions published by the CMS. For the primary outcome, following the above CMS specification, we defined a patient as one who failed to meet the *Controlling High Blood Pressure* quality measure if (1) BP was not recorded during any visit included in the study period or (2) the last BP recorded during the study period was ≥140/90 mm Hg.

For the primary exposure variable—telemedicine visit use—we defined telemedicine visits based on appointment type (ie, visits that were scheduled and conducted by using video or telephone technology) [2]. Because the distribution of telemedicine visits is right-skewed, we classified patients into the following three categories: those with in-person visits only, those with 1 telemedicine visit, and those with ≥2 telemedicine visits. To adjust for the total number of visits (including in-person and telemedicine visits), we similarly defined the following patient categories: patients with a total number of 1, 2, or ≥3 outpatient visits during the study period.

We used patients’ self-reported race and ethnicity to classify patients into the following race and ethnicity categories: non-Hispanic White; non-Hispanic Black; Hispanic; Asian, Hawaiian, and Pacific Islander; and other, declined to answer, or unknown. Patient insurance was categorized as “commercial,” “Medicare,” or “Medicaid” by using the primary payer field associated with the last visit during the study period. Using a similar approach as the one described above for hypertension, we identified patients with a diagnosis of atherosclerotic cardiovascular disease (ASCVD) and diabetes mellitus through visit-associated ICD-10 codes.

### Statistical Analysis

Descriptive statistics were calculated for the proportion of visits in which BP was recorded (ie, for each type of visit [in-person, video, or telephone visits]). Descriptive statistics were also calculated for demographic and clinical characteristics according to the categories of telemedicine use. A chi-square test, a 1-way ANOVA, and the Kruskal-Wallis test were used for categorical variables, normally distributed continuous variables, and nonnormally distributed continuous variables, respectively. A multivariable logistic regression model was used to determine if telemedicine use was associated with higher odds of poor BP control; we adjusted for age, race and ethnicity, payer, and the presence of comorbidities. Because telemedicine visits frequently do not have recorded BP data, we applied the same model for the subgroup of patients with at least 1 recorded BP. As disparities in telemedicine visit use have been previously described for older patients and by race, ethnicity, and insurance status [2,4,5], we conducted additional subgroup analyses for patients aged ≥65 years, non-Hispanic Black patients, Hispanic patients, and patients with Medicaid. All analyses were carried out by using Stata statistical software (version 16; StataCorp LLC).

## Results

For the study population, we identified 32,727 patients aged between 18 and 85 years who had at least 1 completed outpatient visit to primary care or cardiology clinics at Columbia University Irving Medical Center between February 1, 2020, to December 31, 2020, and who had a diagnosis of hypertension (but not end-stage renal disease, history of kidney transplant, or pregnancy). Of these patients, 20,745 (63.3%) had an in-person visit only, 6878 (21%) had 1 telemedicine visit, and 5104 (15.6%) had ≥2 telemedicine visits. Detailed baseline characteristics are described in Table 1. Specifically, patients with more telemedicine visit use were more likely to be female, be Hispanic or non-Hispanic Black, and have Medicaid insurance. They were also less likely to have ASCVD but more likely to have diabetes mellitus, and they had a higher total number of visits.

**Figure 1:**
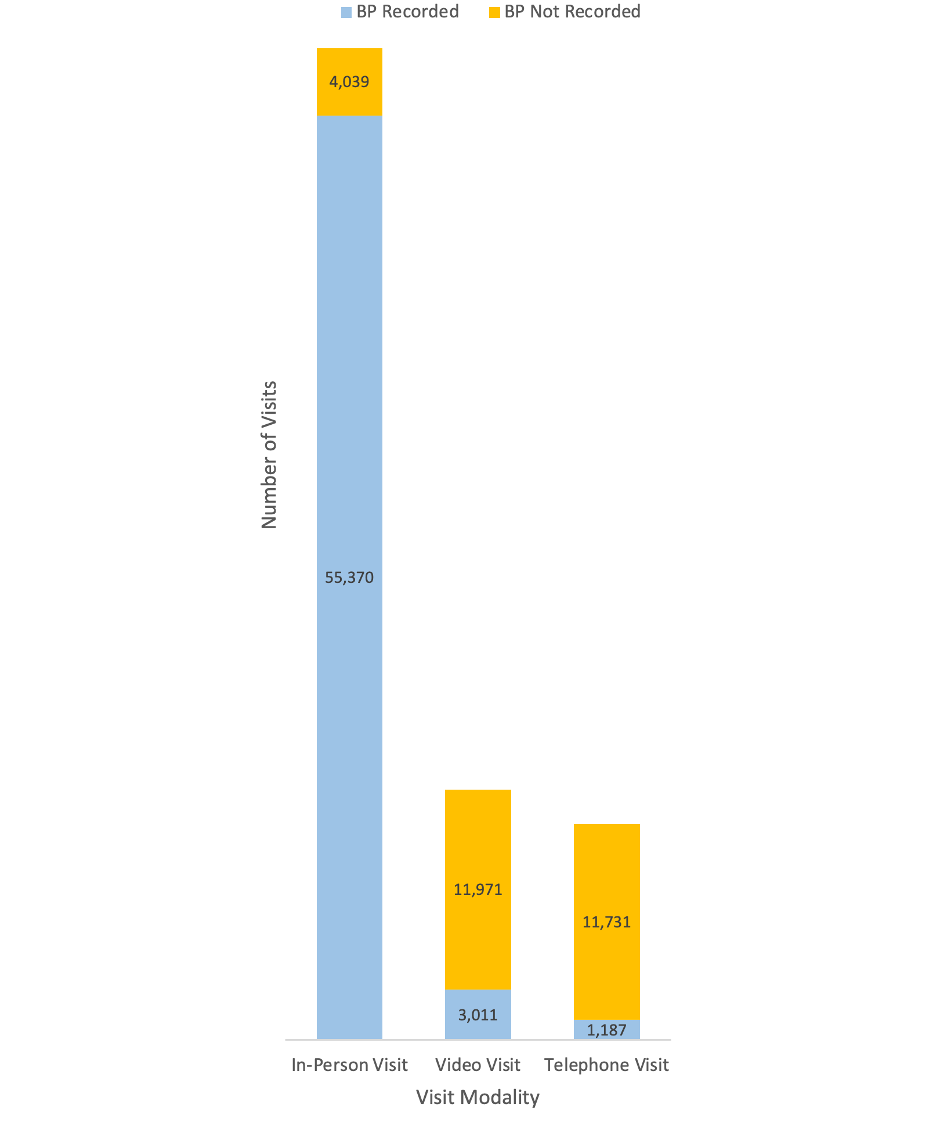
Number of visits by modality (in-person visit, video visit, or telephone visit). For each modality, whether BP was or was not recorded during the visit is also indicated. BP: blood pressure.

Of the total 87,309 visits across the study period, 59,409 were conducted in person, 14,982 were video visits, and 12,918 were telephone visits. BP was recorded for 93% (55,370/59,409) of in-person visits, 20% (3011/14,982) of video visits, and 9% (1187/12,918) of telephone visits (Figure 1).

**Table 1:** Demographic and clinical characteristics of patients with hypertension by telemedicine use.

Characteristic			All in-person visits (n=20,745)		1 telemedicine visit (n=6878)		≥2 telemedicine visits (n=5104)		*P* value	
Age (years), mean (SD)			66.7 (11.8)		65.7 (11.9)		65.4 (12.1)		.08	
**Sex, n (%)**										<.001
	Male	10,489 (50.6)		3325 (48.3)		1868 (36.6)				
	Female	10,256 (49.4)		3553 (51.7)		3236 (63.4)				
**Race and ethnicity, n (%)**										<.001
	Non-Hispanic White	7492 (36.1)		1946 (28.3)		883 (17.3)				
	Hispanic	3127 (15.1)		1810 (26.3)		2484 (48.7)				
	Non-Hispanic Black	1518 (7.3)		600 (8.7)		537 (10.5)				
	Asian, Hawaiian, and Pacific Islander	485 (2.3)		140 (2)		90 (1.8)				
	Other, declined to answer, or unknown	8123 (39.2)		2382 (28.3)		1110 (21.8)				
**Primary insurance, n (%)**										<.001
	Commercial	7169 (34.6)		2150 (31.3)		927 (18.2)				
	Medicare	11,865 (57.2)		3826 (55.6)		2982 (58.4)				
	Medicaid	1711 (8.3)		902 (13.1)		1195 (23.4)				
**Comorbidities, n (%)**										
	Atherosclerotic cardiovascular disease	6375 (30.7)		1990 (28.9)		1257 (24.6)		<.001		
	Diabetes mellitus	3439 (16.6)		1532 (22.3)		1792 (35.1)		<.001		
Total number of visits, median (IQR)			2 (1-3)		2 (1-4)		4 (3-7)		<.001	
**Blood pressure^a^ (mm Hg), mean (SD)**										
	Systolic blood pressure	132.4 (16.3)		132.5 (17.0)		134.3 (17.9)		<.001		
	Diastolic blood pressure	77.5 (9.7)		77.3 (9.7)		78.0 (10.0)		.004		
**Hypertension control status, n (%)**										<.001
	No blood pressure measured	486 (2.3)		1863 (27.1)		1277 (25)				
	Last recorded blood pressure of <140/90 mm Hg	13,374 (64.5)		3346 (48.7)		2384 (46.7)				
	Last recorded blood pressure of ≥140/90 mm Hg	6885 (33.2)		1863 (27.1)		1443 (28.3)				

^a^Calculated from patients with at least 1 recorded BP.

In a multivariable model that was adjusted for demographic and clinical characteristics, telemedicine use was associated with higher odds of not meeting the *Controlling High Blood Pressure* quality measure (1 telemedicine visit: odds ratio [OR] 2.06, 95% CI 1.94-2.18; *P*<.001; ≥2 telemedicine visits: OR 2.49, 95% CI 2.31-2.68; *P*<.001; reference: in-person visits only; Table 2). Older age, Hispanic or Black patients, Medicaid insurance, and diabetes mellitus were also associated with higher odds of not meeting the measure, while ASCVD and 2 or ≥3 total visits during the study period were associated with lower odds of not meeting the measure.

**Table 2:** Multivariable analysis for predictors of the failure to meet the Controlling High Blood Pressure (BP) quality measure, including all patients with hypertension and only those with at least 1 recorded BP. The failure to meet the measure is defined as having (1) no BP recorded at any visit or (2) a last recorded BP of ≥140/90 mm Hg.

Predictors		All patients with hypertension (n=32,727)		Patients with at least 1 recorded BP (n=29,101)	
		Odds ratio (95% CI)	*P* value	Odds ratio (95% CI)	*P* value
Age (per 10-year increase)		1.03 (1.00-1.05)	.04	1.02 (0.99-1.05)	.14
**Sex**					
	Male	Reference	N/A^a^	Reference	N/A
	Female	1.03 (0.99-1.08)	.18	1.07 (1.02-1.13)	.009
**Race and ethnicity**					
	Non-Hispanic White	Reference	N/A	Reference	N/A
	Hispanic	1.45 (1.36-1.55)	<.001	1.43 (1.33-1.54)	<.001
	Non-Hispanic Black	1.58 (1.45-1.73)	<.001	1.63 (1.48-1.79)	<.001
	Asian, Hawaiian, and Pacific Islander	0.97 (0.82-1.13)	.67	0.96 (0.81-1.14)	.65
	Other, declined to answer, or unknown	1.03 (0.97-1.09)	.29	1.05 (0.99-1.12)	.10
**Primary insurance**					
	Commercial	Reference	N/A	Reference	N/A
	Medicare	0.97 (0.91-1.04)	.36	1.00 (0.94-1.08)	.94
	Medicaid	1.28 (1.18-1.38)	<.001	1.28 (1.17-1.39)	<.001
**Comorbidities**					
	Atherosclerotic cardiovascular disease	0.71 (0.68-0.75)	<.001	0.72 (0.68-0.76)	<.001
	Diabetes mellitus	1.10 (1.04-1.16)	.002	1.12 (1.05-1.19)	<.001
**Total number of visits**					
	1	Reference	N/A	Reference	N/A
	2	0.72 (0.69-0.77)	<.001	1.09 (1.02-1.16)	.01
	≥3	0.49 (0.46-0.52)	<.001	1.11 (1.04-1.19)	.002
**Number of telemedicine visits**					
	In-person visit only	Reference	N/A	Reference	N/A
	1 telemedicine visit	2.06 (1.94-2.18)	<.001	0.89 (0.83-0.95)	.001
	≥2 telemedicine visits	2.49 (2.31-2.68)	<.001	0.91 (0.83-0.99)	.03

^a^N/A: not applicable.

When restricting the analysis to the 29,101 patients (patients with in-person visits only: n=20,259, 69.6%; patients with 1 telemedicine visit: n=5015, 17.2%; patients with ≥2 telemedicine visits: n=3825, 13.1%) with a least 1 recorded BP, telemedicine use was associated with lower odds of not meeting the *Controlling High Blood Pressure* quality measure (1 telemedicine visit: OR 0.89, 95% CI 0.83-0.95; *P*<.001; ≥2 telemedicine visits: OR 0.91, 95% CI 0.83-0.99; *P*=.03; reference, in-person visit only). In this model, female sex, Hispanic patients of color, Black patients, Medicaid insurance, and diabetes mellitus were associated with higher odds of not meeting the measure, while ASCVD and 2 or ≥3 total visits during the study period continued to be associated with lower odds of not meeting the measure (Table 2).

Subgroups analyses for patients who were aged ≥65 years, Hispanic patients, non-Hispanic Black patients, and those with Medicaid insurance are shown in Multimedia Appendix 1. The impact of telemedicine use on BP control in these subgroups was similar to that in the analysis that was restricted to the subgroup of patients with at least 1 recorded BP, both in the model that included all patients with hypertension and when only patients with at least 1 recorded BP were included.

## Discussion

In our analysis of patients with hypertension who were seen in primary care and cardiology clinics at an urban, academic medical center during the COVID-19 pandemic in 2020, we found that increased telemedicine visit use was associated with poorer performance on the *Controlling High Blood Pressure* quality measure. These findings are largely driven by BP being recorded in less than 20% (4198/27,900) of telemedicine visits, as BP was recorded in 93% (55,370/59,409) of in-person visits. When the analysis was restricted to patients with at least 1 recorded BP, patients with higher telemedicine visit use had a better or similar likelihood of poor performance on the *Controlling High Blood Pressure* quality measure. These findings were also robust for the subgroups of patients who were previously described to have more difficulty with using telemedicine services, including patients aged ≥65 years, Black or Hispanic patients, and those with Medicaid insurance [2,4,5,17,18].

Our finding that primary care and cardiology telemedicine visits are less likely to have recorded B*P* values when compared to in-person visits is consistent with prior literature. A recent report from a large US database containing 125.8 million primary care visits from 2018 to 2020 demonstrated that BP is recorded in less than 10% of telemedicine visits, whereas BP is recorded in approximately 70% of in-person visits [10]. In our analysis, we additionally found that the disparate rate of BP being recorded at telemedicine visits versus in-person visits resulted in patients who used telemedicine visits being more likely to not meet the *Controlling High Blood Pressure* quality measure. These findings highlight potential unintended consequence of the rapid adoption of telemedicine visits and have direct implications for various quality payment programs, such as those of Medicare accountable care organizations, that use BP control as a key quality benchmark [16]. More broadly, our findings highlight the importance of the continued assessment of the content and quality of care delivered via telemedicine services [10,11], especially as telemedicine visits are expected to remain an integral part of the health care delivery landscape after the COVID-19 pandemic ends.

Nonetheless, it is reassuring that telemedicine visit use did not negatively impact the *Controlling High Blood Pressure* quality measure when the analysis was restricted to patients with at least 1 recorded BP. This suggests that while BP is less likely to be recorded during telemedicine visits, in general the quality of BP management in primary care and cardiology settings may be similar regardless of telemedicine visit use. It is also reassuring that we observed similar results even in populations that are known to have decreased telemedicine use and poorer BP control, such as older patients, Black or Hispanic patients, or patients with Medicaid [2,4,5,19]. However, we cannot fully exclude residual confounding, such as from patients who use more telemedicine services also being more likely to maintain the continuity of care and other health behaviors during the COVID-19 pandemic [6,7,20]. Future studies would need to more rigorously evaluate how telemedicine use can impact BP management as well as determine the best approaches to incorporating BP management strategies, including BP telemonitoring, into routine clinical practice during the telemedicine era [21,22]. Examples of such strategies might include conducting randomized clinical trials of interventions to improve the accuracy of home BP assessments performed by patients in advance of telemedicine and office visits [23] as well as using approaches to integrating telemedicine visits as part of novel BP telemonitoring and medication titration programs [24,25].

There are several additional limitations to our study. As a retrospective cohort study that uses EHR data, our study is necessarily hypothesis-generating research, and additional confounding factors, including those outside of EHR data capture, cannot be excluded. Because we did not assess the quality of BP medications and potential clinical inertia, we have limited insight on how telemedicine use can affect clinical management practices for hypertension. Furthermore, the BP recordings used for this analysis reflect actual clinical practice, and we could not assess the quality of BP measurements that were taken at patents’ homes and then recorded during telemedicine visits, although home BP measurements have been shown to be potentially more reliable than BPs measured during office visits [23,26]. The *Controlling High Blood Pressure* quality measure may also not accurately reflect a patient’s true BP control status at a given point in time. Finally, because there were dramatic care disruptions during the COVID-19 pandemic, our findings may not be generalizable to in-person visits and telemedicine visits when the COVID-19 pandemic ends, and longitudinal research is needed to assess the continued impact of telemedicine visits on cardiovascular care delivery.

Despite these limitations, our study is among the first to describe the real-world impact of telemedicine visits on BP control for a diverse population of patients who access ambulatory care. We found that while the higher use of telemedicine visits was associated with poorer performance on the *Controlling High Blood Pressure* quality measure, this was mainly driven by BP being much less likely to be recorded during telemedicine visits. When BP was recorded, telemedicine use was found to be associated with similar or slightly improved BP control. These results provide timely insights into the impact of telemedicine on cardiovascular care quality with important implications for research, implementation, and policy making in the telemedicine era.

## Appendix

Multimedia Appendix 1 Subgroup analyses on the association between telemedicine use and the failure to meet the “Controlling High Blood Pressure” quality measure for patient populations with potential technology barriers (age: ≥65 years; primary insurance: Medicaid; race and ethnicity: Hispanic people of color and non-Hispanic Black). Poor BP control is defined as having no BP recorded at any visit or having a last recorded BP of ≥140/90 mm Hg. BP: blood pressure; OR: odds ratio.

## Abbreviations

ASCVD: atherosclerotic cardiovascular disease
BP: blood pressure
CMS: Centers for Medicare & Medicaid Services
EHR: electronic health record
ICD-10: International Classification of Diseases, 10th Revision
NHLBI: National Heart, Lung, and Blood Institute
OR: odds ratio
