# Supplementary material for: The Impact of Telemedicine Visits on the Controlling High Blood Pressure Quality Measure During the COVID-19 Pandemic: Retrospective Cohort Study
Source: JMIR Form Res. 2022 Mar 23;6(3):e32403. doi: 10.2196/32403 (PMC8945081; doi:10.2196/32403)
Supplement: Multimedia Appendix 1 [file formative_v6i3e32403_app1.docx]

**Multimedia Appendix 1.** Subgroup analyses on association between telemedicine use and failure to meet the Controlling High Blood Pressure quality measure for patient populations with potential technology barriers (age ≥ 65 years old, Medicaid, non-White Hispanic, and non-Hispanic Black). Poor BP control is defined as having no BP recorded at any visit OR last recorded BP ≥ 140/90 mm/Hg. OR: odds ratio; CI: confidence interval.

| **Number of Telemedicine Visits** | **Age ≥ 65 years old** | | | | **Medicaid** | | | |
| --- | --- | --- | --- | --- | --- | --- | --- | --- |
|  | **All Patients**  **(n=19,591)** | | **With BP Recorded**  **(n=17,644)** | | **All Patients**  **(n=3,808)** | | **With BP Recorded**  **(n=3,150)** | |
|  | **OR (95% CI)** | ***p*-value** | **OR (95% CI)** | ***p*-value** | **OR (95% CI)** | ***p*-value** | **OR (95% CI)** | ***p*-value** |
| In-person visit only  1 telemedicine visit  2+ telemedicine visits | Ref  2.06 (1.94 to 2.18)  2.49 (2.31 to 2.68) | Ref  <.001  <.001 | Ref  0.90 (0.82 to 0.98)  0.95 (0.85 to 1.06) | Ref  .02  .32 | Ref  2.39 (2.00 to 2.85)  2.66 (2.21 to 3.19) | Ref  <.001  <.001 | Ref  1.04 (0.85 to 1.28)  0.88 (0.72 to 1.09) | Ref  .69  .24 |
|  | **Hispanic, non-White** | | | | **Black, non-Hispanic** | | | |
|  | **All Patients**  **(n=7,421)** | | **With BP Recorded**  **(n=6,167)** | | **All Patients**  **(n=2,655)** | | **With BP Recorded**  **(n=2,319)** | |
|  | **OR (95% CI)** | ***p*-value** | **OR (95% CI)** | ***p*-value** | **OR (95% CI)** | ***p*-value** | **OR (95% CI)** | ***p*-value** |
| In-person visit only  1 telemedicine visit  2+ telemedicine visits | Ref  2.27 (2.01 to 2.57)  2.67 (2.35 to 3.02) | Ref  <.001  <.001 | Ref  0.94 (0.82 to 1.09)  0.89 (0.77 to 1.03) | Ref  .44  .11 | Ref  1.91 (1.57 to 2.33)  2.56 (2.04 to 3.23) | Ref  <.001  <.001 | Ref  0.82 (0.65 to 1.04)  1.03 (0.79 to 1.34) | Ref  .11  .82 |
